# Antiretroviral treatment adherence among HIV patients in KwaZulu-Natal, South Africa

**DOI:** 10.1186/1471-2458-10-111

**Published:** 2010-03-05

**Authors:** Karl Peltzer, Natalie Friend-du Preez, Shandir Ramlagan, Jane Anderson

**Affiliations:** 1 Health Systems Research Unit, Social Aspect of HIV/AIDS and Health, Human Sciences Research Council, Pretoria, South Africa; 2 Department of Psychology, University of the Free State, Bloemfontein, South Africa; 3 Centre for Population Studies, London School of Hygiene and Tropical Medicine, London, UK; 4 Centre for the study of Sexual Health and HIV, Homerton University Hospital NHS Foundation Trust, London, UK

## Abstract

**Background:**

Successful antiretroviral treatment is dependent on sustaining high rates of adherence. In the southern African context, only a handful of studies (both quantitative and qualitative) have looked at the determinants including a health behaviour theory of adherence to antiretroviral therapy. The aim of this study is to assess factors including the information, motivation and behavioural skills model (IMB) contributing to antiretroviral (ARV) adherence six months after commencing ARVs at three public hospitals in KwaZulu-Natal, South Africa.

**Methods:**

Using systematic sampling, 735 HIV-positive patients were selected prior to commencing on ART from outpatient departments from three hospitals and followed-up at six months and interviewed with a questionnaire.

**Results:**

A good proportion of patients were found to be adherent using both adherence instruments (visual analog scale = VAS 82.9%; Adult AIDS Clinical Trials Group = AATCG 70.8%). After adjusting for significant socio-economic variables, both the VAS and the dose, schedule and food adherence indicator found levels of adherence amongst urban residents to be almost 3 times greater than that of rural residents. After adjusting for health-related variables, for both indicators better adherence was associated with low depression and poorer adherence was associated with poor environmental factors. Adjusted odds ratios for adherence when taking into account different behavioural variables were for both adherence indicators, discrimination experiences were associated with lower adherence, and higher scores in adherence information and behavioural skills were associated with higher adherence. For the VAS adherence indicator, higher social support scores were associated with higher adherence. For the dose, schedule and food adherence indicator, using herbal medicines for HIV was associated with lower adherence.

**Conclusion:**

For the patients in this study, particularly those not living in urban areas, additional support may be needed to ensure patients are able to attend appointments or obtain their medications more easily. Adherence information and behavioural skills as part of the IMB model should be strengthened to improve adherence. Further psychological support is also required and patients' perceived need for ARTs should be routinely assessed.

## Background

The clinical efficacy of antiretroviral therapies (ART) in suppressing the HIV virus and improving survival rates for those living with HIV has been well documented [1-5]. However, successful antiretroviral treatment is dependent on sustaining high rates of adherence (correct dosage, taken on time and in the correct way - either with or without food). The minimum level of adherence required for antiretrovirals (ARVs) to work effectively is 95% [6]. Although more potent ARV regimens can allow for effective viral suppression at moderate levels of adherence [7-9], non or partial adherence can lead to the development of drug-resistant strains of the virus. In resource-limited settings where older first-line therapies are being used, the development and transmission of drug-resistant strains of HIV will greatly limit the treatment options available.

A meta-analysis conducted by Mills et al. [10], examined barriers and facilitators of ART adherence in 72 developed and 12 developing country settings (5 African). Barriers to adherence in both settings included fear of disclosure, forgetfulness, health illiteracy, substance abuse, complicated regimens, and patients being away from their medications. In developing settings, financial constraints and a disruption in access to medications were also common barriers. Other factors known to affect adherence include issues related to gender [11,12] and stigma [13-15]. In the southern African context, only a handful of studies (both quantitative and qualitative) have looked at the determinants of adherence to antiretroviral therapy. Common barriers identified include fear of disclosure, alcohol use, traditional medicine use, feeling better on treatment, inadequate knowledge about the disease and ARVs, stigma, transport costs, [16-20], lack of social support (financial and emotional) [17], stigma, discrimination, depression and hopelessness, not being able to disclose their HIV status and a lack of food [19,20], service-related factors [18,20], patients' beliefs and behaviours [18], pill burden and drug side-effects [18,20].

There is a lack of studies investigating treatment competency factors and also utilizing a health behaviour theory such as the Health Belief Model [18] in relation to ART adherence in Africa [21]. One promising health behaviour theory that has been tailored specifically to designing interventions to promote adherence to ART in developed countries is the information, motivation and behavioural skills model (IMB) [22]. The aim of this study is to assess factors including the information, motivation and behavioural skills model (IMB) contributing to ARV adherence six months after commencing ARVs at three public hospitals in KwaZulu-Natal, South Africa.

## Methods

### Design and setting

This is a cross-sectional study of all treatment-naúve patients (N = 735) recruited from all three public hospitals in Uthukela health district in KwaZulu-Natal from October 2007 to February 2008. The District has one regional and two district hospitals, one private hospital, three primary health care facilities, 24 fixed clinics and 17 mobile clinics with 177 visiting points [23]. Initiation to ART is done at the three public hospitals. Some patients are referred to primary care clinics for ARV collection but return to the hospital for six monthly visits. HIV treatment is provided free of charge. The treatment programme provides patients with access to counselling, nutritional assistance, psychosocial support and social welfare evaluation.

### Sample and procedure

All ARV-naúve patients who were about to commence ARVs (18 years and above) and who consecutively attended the HIV clinics during the recruitment period were eligible for this study. Physicians from the three selected public clinics asked every consecutively visiting ART-naïve patient meeting the inclusion criteria of being 18 years or over if they would like to complete a confidential survey and interview concerning their health and social situation. This would include information from their medical records on details of their medical condition, laboratory tests and treatment. If the potential participant indicated an interest in participating, the health care provider then referred them to an external HSRC research assistant for possible research participation. The interviews were conducted by four trained external HSRC researchers (one or two per HIV clinic) in interview administration of the semi-structure interview schedule. Interviewers were trained over one week in questionnaire administration and ethics procedures. Recruitment took place over a period of four months, with 97.8% participation rate. The questionnaire was translated into the major language spoken in the study area (Zulu) and verified by a second translator. Where inconsistencies were found, these were corrected. Pre-testing of the questionnaire was completed with five HIV-positive persons not involved in the study. More details about the setting, sampling procedure and recruitment have been described elsewhere [24]. Patients at six months follow-up were interviewed at the clinic. Patients who failed to attend for planned follow-up were contacted by telephone and up to two home visits.

Ethics approval was obtained from the HSRC ethics committee and approval was obtained from the Provincial Department of Health in KwaZulu-Natal.

### Measures

Patients were interviewed with an anonymous questionnaire that requests information on sociodemographic characteristics, clinical history and health-related characteristics and health beliefs. Clinical data relating to date of HIV diagnosis, HIV acquisition and transmission risk factors, current CD4 cell count, viral load (Chiron 3.0 bDNA), opportunistic infections, HIV and non-HIV medications was obtained from the medical chart.

#### The Revised Sign and Symptom Checklist for Persons with HIV Disease

The SSC-HIVrev is a 72-item checklist of HIV/AIDS specific physical and psychological symptoms, scored using the following scale: 0 = not present today, 1 = mild, 2 = moderate, 3 = severe [25]. Female-specific symptoms were removed, reducing the total to 64 [24]. An HIV symptom index (symptom intensity) was created which weights each symptom's presence (0 or 1) by a rating of 1-3 (mild, moderate or severe). Cronbach's alpha of this scale for this sample was 0.84.

#### Health-Related Quality of Life

The WHOQOL-HIV BREF is based on the WHOQOLHIV measure, one of the two World Health Organization's QoL instruments for use with HIV-infected populations [26]. This instrument is intended for cross-cultural use and is meant to be accessible to researchers in low-income countries. The individual respondent's overall QoL are measured directly: 'How would you rate your quality of life?' (ranging from 'very poor' to 'very good'); 'How satisfied are you with your health?' (ranging from 'very dissatisfied' to 'very satisfied'). The 31-item WHOQOLHIV BREF produces six domain scores, which denote an individual's subjective perception of their own QoL in the following domains: physical, psychological, level of independence, social relationships, physical environment and spirituality. The individual items are rated on a 5-point Likert scale where '1' indicates 'low, negative perceptions' and '5' indicates 'high, positive perceptions.' Domain scores are scaled in a positive direction, where higher scores denote higher perceived QoL [27]. Reliability was good for five of the six domains (Cronbach's alpha 0.60-0.72) and lower for the social relationships domain (Cronbach's alpha 0.46). Cronbach's alpha for the whole HRQoL scale was 0.88 for this sample.

#### Alcohol Use Disorder

Identification Test (AUDIT)-C focuses solely upon consumption of alcohol (i.e. the frequency of drinking, the quantity consumed at a typical occasion, and the frequency of heavy episodic drinking (i.e. consumption of six standard drinks or more on a single occasion - in South Africa a standard drink is 12 g alcohol) [28]. Because AUDIT is reported to be less sensitive at identifying risk drinking in women [29], the cut-off points of binge drinking for women were reduced by one unit as compared with men. Gual et al. [30] recommend a cut-off point of ≥ 5 for men and ≥ 4 for women although the false-positive rate was 46.5% among male and 63.3% among female patients when compared with a clinical diagnosis of risky drinking. Cronbach's alpha for the AUDIT-C in this sample was 0.85.

#### Internalized AIDS stigma

Items were adapted to assess internalized AIDS stigma from a scale developed to measure AIDS related stigma beliefs in general South African populations. We selected seven items from the AIDS-Related Stigma Scale [31] and reframed the wording to represent negative self-perceptions and self-abasement in relation to being a person living with HIV/AIDS. The items focused on self-blame (e.g., "I sometimes feel worthless because I am HIV positive.") and concealment of HIV status from others (e.g., "I hide my HIV status from others."). In this study, we examined responses to each of the four internalized stigma items as individual indicators of internalized AIDS stigma and we computed a scale by summing all items endorsed in the direction of greater internalized stigma. Items were responded to from 1 = strongly agree to 4 = strongly disagree. Strongly agree and agree were converted to "1" and strongly disagree and disagree to "0"; scale scores represent the sum total of endorsed items, range 0-7. Cronbach's alpha for this stigma index was 0.64 for this sample.

#### HIV/AIDS discrimination experiences

To assess AIDS-related discrimination, we asked participants if they had experienced seven discrimination-related events, e.g., whether they had been treated differently since they had disclosed their HIV status to friends and family; whether being HIV positive had caused them to lose a job or a place to stay; and whether they had experienced discrimination because they are HIV positive. Response options were "yes" or "no". Cronbach's alpha for this sample was 0.54.

#### Social support

Three items were drawn from the Social Support Questionnaire to assess perceived social support [32]. The items were selected to reflect perceived tangible and emotional support: If I were sick and needed someone to take me to a doctor I would have trouble finding someone (reversed); I feel that there is no one I can share my most private concerns and fears (reversed); and I feel a strong emotional bond with at least one other person. These items were responded to on 4-point scales, 1 = completely true, to 4 = completely false, and summed to a score with a range of 3-12. Cronbach's alpha for this sample was .83.

We assessed *depressive symptoms *using the 10-item version of the Centers for Epidemiologic Studies Depression Scale (CES-D) [33]. The CES-D has been widely used in studies of the relationship between HIV and depression [34]. Cronbach's alpha for this sample was 0.54.

#### Adherence assessment

ARV treatment adherence was assessed by two self-reported adherence measures - the Adult AIDS Clinical Trials Group (AACTG) adherence instrument and the 30-day visual analog scale (VAS). The AACTG consists of nine questions that assess adherence from the previous 1-4 days, within the past week, prior to the interview. The instrument also assesses reasons for non-adherence [35]. The 30-day visual analog scale (VAS) provided an overall adherence assessment for a longer time interval (one month). Both have been validated in resource-limited settings [36,37]. Adherence is calculated as the % of doses taken over those prescribed. Adherence levels assessed from the VAS are defined as follows: full adherence = 100%, partial adherence >/= 95% and < 100%, and non-adherence as < 95% of prescribed doses taken since the last refill.

Dose adherence was assessed by asking participants to report on how many days they had missed taking all their doses during the past 4 days. Dose non-adherence was defined as having missed all doses on at least one day during the past 4 days.

Adherence to scheduling was measured by the question "Most anti-HIV medications need to be taken on a schedule, such as '2 times a day' or '3 times a day' or 'every 8 hours.' The participants were asked to report how closely they followed their specific schedule over the last 4 days using a 5-point Likert scale, ranging from "never" to "all the time." Schedule non-adherence was defined as having missed scheduling in the past 4 days. Adherence to dietary instructions was measured by first asking "Do any of your anti-HIV medications have special food instructions, such as 'take with food' or 'on an empty stomach' or 'with plenty of fluids'?" If the response was "yes," participants were asked to rate how often they had followed dietary instructions over the last 4 days using a 5-point Likert scale, ranging from never" to "all the time." Schedule non-adherence was defined as having missed scheduling in the past 4 days. Food non-adherence was defined as not having followed special instructions over the last 4 days.

The *LifeWindows Information-Motivation-Behavioural Skills ART adherence questionnaire *(LW-IMB-AAQ) [38,39]. Each LW-IMB-AAQ item represents a barrier primarily falling within the I (Information), M (Motivation), or B (Behavioural Skills) constructs. Adherence information was assessed with five items (α .69). Example for an information item: "I know what to do if I miss a dose of any of my HIV medications (for example, whether or not to take the pill(s) late)." Responses to items include "yes," "no," or "don't know" ("don't know" responses were keyed as incorrect responses). Adherence motivation was assessed with ten items (α .78). A "motivation" sample item: "I am worried that other people might realize that I am HIV+ if they see me taking my HIV medications." Response options were 1 = strongly disagree to 5 = strongly agree. Behavioural skills were assessed with 14 items (α .73). An example of a behavioural skills item: "How hard or easy is it for you to stay informed about HIV treatment?" Response options were 1 = cannot do at all to 5 = certain you can do.

### Data analysis

Data were analyzed using Statistical Package for the Social Sciences (SPSS) for Windows software application programme version 17.0. Frequencies, means, standard deviations, median, interquartile range, were calculated to describe the sample. Uni- and bi-variate analyses and, multiple logistic regressions were used to investigate associations between the outcomes ART adherence and socioeconomic variables, health related variables, and behavioural variables as well as information-mativation-behavioural skills model variables. Associations were considered significant at P < 0.05. Separate multivariable logistic regression analyses were conducted for socio-demographic variables, health related variables and behavioural variables (moderating factors) and information-mativation-behavioural skills model variables and ART adherence. All variables statistically significant at the P < .01 level in bivariate analyses were included in the multivariate model. No significant interactions were found between socioeconomic variables, health related variables, behavioural variables and information-mativation-behavioural skills model variables.

## Results

### Sample characteristics

Of 735 patients (29.8% male and 70.2% female) who completed assessments prior to initiation of ARVs, 525 were able to complete the assessment at six months follow-up. Of the original cohort, 75 had died, 57 had been transferred, 54 could not be traced, 23 refused the interview and 1 interview was incomplete. At six months following proposed ARV initiation, 519 patients started therapy and six failed to start treatment. Over the six month period 24 patients (4.6%) had temporarily suspended ARVs because of side effects, and three (0.6%) had changed their ARVs. HIV medications for 411 (79.2%) patients included Lamivudine (3TC), Stavudine (d4T) + Efavirenz (Stocrin) and for 108 (20.8%) Lamivudine (3TC), Stavudine (d4T) + Nevirapine. Fixed dose combination of ARVs was not available for patients on this programme during the time of the study.

Nearly three-quarters (73.5%) of the 519 patients who had initiated ARVs in this sample were female, 62.2% of whom were between 30 and 49 years old. Nearly three-quarters (73.3%) were never married, 61.9% had Grade 8 or higher formal education, almost all (98.8%) were Zulu and the largest religious affiliation was charismatic churches (38.5%). The majority of the sample (61.7%) lived in rural areas and was unemployed (59.6%). Only 31.7% of respondents had a formal salary as their main source of household income and 52.5% was in receipt of a disability grant. Those who were followed up at six months (n = 525) were compared to those who could not be followed up (n = 210) on sex, age, formal education, urban or rural residence, HIV symptoms, CD4 cell count and in recept of a disability grant. We found that those who could not be followed up were more likely to be male (χ^2 ^= 8.13, P = .004) and had a lower CD4 cell count (t = -2.55, p = .011) (*v*. Table 1).

**Table 1:** Sample characteristics

Variable	N = 519	%
***Sex***		
Male	139	26.6
Female	370	73.4

***Age in years***	136	26.3
18-29	222	42.9
30-39	100	19.3
40-49	59	11.4
50 and above		

***Marital status***		
Never married	379	73.3
Currently married	68	13.2
Cohabitating	40	7.7
Divorced/separated	11	2.2
Widowed	19	3.7

***Highest education***		
None	40	7.7
Up to Grade 7	157	30.4
Grade 8-11	221	42.7
Grade 12 or more	99	19.1

***Ethnicity***		
Zulu	513	98.8
Other	6	1.2

***Religious affiliation***		
African/traditional	52	10.0
Christian (Protestant churches)	73	14.1
Christian (Catholic)	49	9.4
Apostolic	48	9.2
Zion Christian Church	152	29.3
Other	71	14.0
No religion	74	14.3

***Residence***		
Rural village	221	42.7
Informal settlements (slums)	31	6.0
Urban/metropolitan areas	49	9.5
Township	118	22.8
Farm	98	19.0

***Employment situation***		
Housewife, home maker	76	15.0
Unemployed	303	59.6
Employed	115	22.6
Pensioner, student, disabled	21	4.2

***Main source of household income***		
Formal salary	162	31.7
Contribution by family members	86	16.9
Government grant	113	22.1
Grants/donations by private welfare organizations	80	15.7
No income (other than social grant)	38	7.4
Other	32	6.3

***Disability grant ("for HIV/AIDS")***		
Yes	268	52.5
No	242	47.5

### Health characteristics

Most patients (75.2%) had been diagnosed with HIV in the year prior to study recruitment. The median CD4 count at follow-up was 130 cells/cu.mm compared to 119 cells/cu.mm prior to ARV initiation. The mean number of HIV symptoms reported at follow-up was 1.21, 6.6% of patients were receiving TB treatment, 10.3% had at least one hospital admission in the past six months, and 25.6% had seen an ARV treatment buddy at least once in the past six months. Patients with an identified adherence problem are referred to a treatment buddy (*v*. Table 2).

**Table 2:** Health and behavioral characteristics

Variable	N = 519	%
**Time since HIV diagnosis**		
2007/8	379	75.2
2006-1995	125	24.8

**CD4 count (cells/uL) = Median = **130 (IQR = 72-185) (at baseline: Median = 119; IQR = 59-163)		
1-99	188	37.2
100-200	232	45.9
>200	85	16.8

**Number of HIV symptoms **(range 0-20) M(SD)	1.21 (2.60)	

**Overall Quality of Life (range 1-5) M(SD)**	4.3 (0.7)	

**General health perceptions (range 1-5) M(SD)**	4.4(0.7)	

**Depression score (range 10-40) M(SD)**	17.3(3.3)	

**Receiving TB treatment**	34	6.6

**Hospital admission in the past 6 months**	53	10.3

**Participated in support group in the past 6 months**	14	2.7

**Seen someone for counseling/support in the past 6 months**	123	23.8

**Seen an ARV treatment buddy in the past 6 months**	132*	25.6

**Had alcohol in the past month**	13	2.5

**AUDIT-C 4 or more**	10	1.9

**Stigma score (range 0-7) M(SD)**	2.8 (1.8)	

**Discrimination experience score (range 0-5) M(SD)**	0.87 (1.11)	

**Social support score (range 3-12) M(SD)**	8.3 (2.3)	

*Of those who saw a treatment buddy, 93% saw a buddy only once in the past 6 months

### ART adherence

Using the 30-day visual analog scale (VAS) 427 patients (82.9%) were 95% adherent in the month prior to the survey. Results from the AACTG adherence instrument found that on the 4-day recall dose adherence, 15.5% of patients were non-adherent (having missed at least one full day of medication in the past four days). 70.8% of patients were adherent to all parameters (dose, schedule and food). Pearson correlation among the two adherence outcome measures (VAS and AACTG) using categorical cutoffs to define adherence indicated a moderate level of association (*r *= .56, *P *< .001). From those found non-adherent on the VAS (17.1%) 85.2% were also found to be non-adherent on the AACTG measure (*v*. Table 3).

**Table 3:** ART adherence

		n	%
30-day VAS at 95%	Adherent	427	82.9
	Non-adherent	88	17.1
			
Self-reported 4-day recall dose adherence	Adherent	435	84.5
	Non-adherent	80	15.5
			
Self-reported time adherence	Adherent	372	72.4
	Non-adherent	142	27.6
			
Self-reported food adherence	Adherent	369	71.7
	Non-adherent	146	28.3
			
Adherence to all (Dose, Schedule and Food)	Adherent	364	70.8
	Non-adherent	150	29.2

### Determinants of ART adherence

Both the VAS and the dose, schedule and food adherence indicator found levels of adherence amongst urban residents to be almost 3 times greater than that of rural residents. The VAS indicator found greater adherence amongst those with lower levels of education and amongst single, separated, divorced or widowed groups compared to those married and cohabiting (*v*. Table 4).

**Table 4:** Association between socioeconomic variables and ART adherence

	VAS adherence (≥ 95%)			Dose, schedule and food adherence	
	**N = 519 (%)**	**Crude OR** **(95% CI)**	**P**	**Crude OR** **(95% CI)**	**P**

*Sex*					
Female	370 (26.6)	1.00		1.00	
Male	139 (73.4)	1.08 (0.54-1.56)	.747	1.03 (0.63-1.50)	.887

Age		0.99 (0.97-1.02)	.673	1.01 (0.99-1.03)	.615

*Formal education*					
lower (up to Grade 7)	197 (38.1)	1.00		1.00	
higher (Grade 8 or more)	320 (61.9)	0.89 (0.81-1.00)	.050	0.99 (0.91-1.08)	.895

*Marital status*					
Married/cohabitating	108 (20.9)	1.00		1.00	
Single/separated/divorced/widowed	409 (79.2)	1.79 (1.02-3.00)	.028	0.99 (0.62-1.59)	.974

*Employment status*					
Unemployed	393 (77.4)	1.00		1.00	
Employed	115 (22.6)	1.00 (0.58-1.74)	.991	1.23 (0.77-1.97)	.386

*On disability grant ("for AIDS")*					
No	242 (47.5)	1.00		1.00	
Yes	268 (52.5)	1.09 (0.69-1.72)	.722	0.91 (0.62-1.34)	.642

*Residence*					
Rural	319 (61.7)	1.00		1.00	
Urbanct	198 (38.3)	2.78 (1.60-4.83)	.000	3.34 (2.13-5.25)	.000

After adjusting for health-related variables, for both indicators adherence was lower amongst those with higher depression scores and for those with low scores in the Environment domain (safety/healthy physical environment/enough money/access to information/opportunity for leisure activities/transport/access to health services). The dose, schedule and food adherence indicator found adherence to be 3.3 times greater amongst patients with a CD4 count above 200 cells/uL, 4.6 times greater among patients with the 3TC, d4T + Nevirapine regimen and higher overall quality of life. The VAS adherence indicator found higher adherence amongst patients with lower scores in the Spirituality/religion/personal beliefs domain, with higher general health perception scores and with higher scores in social relationships domain (v. Table 5).

**Table 5:** Association between health-related variables and ART adherence

		VAS adherence (≥ 95%)				Dose, schedule and food adherence			
	**N (%)** **519**	**Crude OR** **(95% CI)**	**P**	**Adjusted OR^a, b^** **(95% CI)**	**P**	**Crude OR** **(95% CI)**	**P**	**Adjusted OR^a, c^** **(95% CI)**	**P**

*Time since diagnosis*									
2006-1995	125 (24.8)	1.00				1.00			
2007/8	379 (75.2)	0.49 (0.26-0.92)	.026	...		0.80 (0.50-1.26)	.330	---	

Hospital admission in the past 6 months									
No	463 (89.7)	1.00				1.00			
Yes	53 (10.3)	1.51 (0.62-3.69)	.326	...		0.98 (0.51-1.85)	.937	---	

*CD4 count*									
≤ 200	420 (83.2)	1.00		...		1.00		1.00	
>200	85 (16.8)	1.19 (0.63-2.26)	.599			2.90 (1.52-5.53)	.001	3.32 (1.18-9.38)	.023

*HIV symptoms (range 0-20)*									
M (SD)	1.2 (2.6)	1.08 (0.96-1.21)	.213	...		1.06 (0.97-1.15)	.199	---	

*Overall Quality of Life*^d^									
M (SD)	4.3 (0.7)	2.87 (2.00-4.10)	.000	0.94 (0.52-1.68)	.830	3.91 (2.71-5.65)	.000	2.06 (1.07-3.98)	.031

General health perceptions^d^									
M (SD)	4.4 (0.7)	2.69 (1.95-3.73)	.000	1.72 (1.01-2.95)	.047	3.72 (2.62-5.28)	.000	1.57 (0.89-2.79)	.121

*WHOQOL-HIV BREFscores*									
Physical domain^e^	15.6 (2.7)	1.35 (1.22-1.49)	.000	0.94 (0.78-1.13)	.512	1.54 (1.40-1.69)	.000	0.87 (0.70-1.08)	.201
Psychological domain^e^	14.6 (3.2)	1.40 (1.28-1.53)	.000	1.17 (0.97-1.41)	.099	1.65 (1.50-1.81)	.000	1.00 (0.81-1.23)	.970
Level of independence domain^e^	14.5 (1.9)	1.42 (1.29-1.56)	.080	...		1.10 (0.98-1.24)	.103	...	
Social relationships domain^e^	13.4 (2.6)	1.52 (1.37-1.69)	.000	1.14 (1.00-1.30)	.048	1.65 (1.49-1.83)	.000	1.05 (0.89-1.24)	.549
Environment domain^e^	13.9 (2.6)	1.76 (1.56-2.00)	.000	1.56 (1.28-1.89)	.000	2.46 (2.11-2.87)	.000	2.21 (1.71-2.86)	.000
Spirituality/religion/personal beliefs domain^e^	15.3 (2.9)	1.17 (1.08-1.27)	.000	0.76 (0.63-0.91)	.003	1.40 (1.29-1.51)	.000	1.06 (0.89-1.28)	.507

Depression score (higher score = more depressed)	17.3 (3.3)	0.78 (0.72-0.84)	.000	0.88 (0.80-0.96)	.006	0.63 (0.58-0.69)	.000	0.71 (0.62-0.80)	.000

ART regimen									
3TC, d4T + Efavirenz	411 (79.2)	1.00		1.00		1.00		1.00	
3TC, d4T + Nevirapine	108 (20.8)	4.08 (1.73-9.63)	.001	2.16 (0.80-5.87)	.130	5.25 (2.57-10.72)	.000	4.61 (1.48-14.34)	.008

^a^Using block entry;^b^Hosmer and Lemeshow Chi-square = 11.67, df = 8, p = .166; ^b^Cox & Snell R^2 ^.26; ^b ^Nagelkerke R^2 ^.42;

^c^Hosmer and Lemeshow Chisquare = 25.05, df = 8, p.002; ^c^Cox & Snell R^2 ^.54; ^c ^Nagelkerke R^2 ^.76

^d ^Mean scores range from 1 to 5, with 5 indicating the highest, most positive perceptions of quality of life or general health perceptions.

^e ^Overall domain scores range from 4 to 20, with 20 indicating the highest, most positive perceptions.

Table 6 presents crude and adjusted odds ratios for adherence when taking into account different behavioural variables (moderating) factors and information-motivation-behavioural skills model variables. For both adherence indicators, discrimination experiences were associated with lower adherence, and higher scores in adherence information and behavioural skills were associated with higher adherence. For the VAS adherence indicator, higher social support scores were associated with higher adherence. For the dose, schedule and food adherence indicator, using herbal medicines for HIV was associated with lower adherence (v. Table 6).

**Table 6:** Association between behavioural variables (moderating factors), information-motivation-behavioural skills model and ART adherence

		VAS adherence (≥ 95%)				Dose, schedule and food adherence			
**Behavioural variables (moderating factors)**	**N (%)** **519**	**Crude OR** **(95% CI)**	**P**	**Adjusted OR^a, b^** **(95% CI)**	**P**	**Crude OR** **(95% CI)**	**P**	**Adjusted OR^a, c^** **(95% CI)**	**P**

*TCAM use for HIV*									
No	375 (72.0)	1.00		1.00		1.00			
Yes	144 (28.0)	0.41 (0.28-0.66)	.000	0.62 (0.29-1.34)	.226	0.66 (0.43-1.00)	.049	...	

*Herbal use for HIV*									
No	476 (92.0)	1.00		1.00		1.00		1.00	
Yes	43 (8.0)	0.13 (0.07-0.26)	.000	0.70 (0.21-1.61)	.296	0.04 (0.02-0.12)	.000	0.12 (0.03-0.51)	.004

*Past month alcohol use*									
No	509 (97.5)	1.00		1.00		1.00			
Yes	13 (2.5)	0.08 (0.03-0.28)	.000	0.47 (0.04-5.84)	.556	0.03 (0.00-0.25)	.001	...	

*AUDIT-C 4 or more*									
No	506 (98.1)	1.00		1.00		1.00			
Yes	10 (1.9)	0.08 (0.02-0.33)	.000	0.76 (0.04-13.48)	.851	0.04 (0.01-0.35)	.003	...	

*Discrimination experiences score (higher score= higher level of discrimination)*		0.44 (0.36-0.54)	.000	0.60 (0.46-0.78)	.000	0.20 (0.15-0.27)	.000	0.28 (0.19-0.41)	.000

*Stigma score (higher score= higher stigma)*		1.11 (0.97-1.27)	.141	...		0.98 (0.88-1.09)	.717	...	

*Social support score (higher score = higher support*		1.26 (1.13-1.40)	.000	1.20 (1.00-1.45)	.046	1.17 (1.08-1.28)	.000	0.97 (0.81-1.17)	.769

**Information-motivation-behavioural skills model**									

*IMB adherence information *(higher score = higher adherence information)		1.42 (1.31-1.55)	.000	1.11 (1.01-1.22)	.032	1.55 (1.43-1.69)	.000	1.26 (1.12-1.43)	.000

*IMB adherence motivation *(higher score = higher adherence motivation)		1.13 (1.08-1.17)	.000	1.03 (0.97-1.10)	.333	1.14 (1.10-1.18)	.000	1.02 (0.96-1.10)	.482

*IMB behavioral skills *(higher score = higher behavioural skills)		1.21 (1.16-1.26)	.000	1.07 (1.01-1.14)	.023	1.34 (1.28-1.41)	.000	1.14 (1.07-1.21)	.000

^a ^Using block entry; ^b^Hosmer and Lemeshow Chi-square = 4.93, df = 8, p = .7.65; ^b^Cox & Snell R^2 ^.25; ^b ^Nagelkerke R^2 ^.41;

^c^Hosmer and Lemeshow Chisquare = 13.26, df = 8, p.103; ^c^Cox & Snell R^2 ^.52; ^c ^Nagelkerke R^2 ^.74

## Discussion

A good proportion of patients were found to be adherent using both adherence instruments (VAS 82.9%; AATCG 70.8%). These good figures are similar to that of 77% found for African patients the meta-analysis by Mills and colleagues [40]. Such good rates may however decline the longer patients are on treatment. Kalichman et al. [41] found that the VAS yielded adherence rates that paralleled unannounced pill counts and differed from AATCG recall suggesting that the VAS offers a valid method of assessing medication adherence. However, the combined dose, schedule and food adherence indicator of the AATCG may be useful in identifying schedule and food adherence, and found in this study different adherence rates and influencing factors as compared to the VAS. For example, lower dose, schedule and food adherence was found for patients on 3TC, d4T + Efavirenz regimen and those who were taking herbal medicine for HIV.

Important socio-economic predictors of ART adherence in this South African sample include urban area of residence and adequate physical environment including transport and access to health services. Living in an urban area is likely to be associated with lower transport costs and fewer disruptions in access to medications, which other studies have found to be a facilitators of adherence [11,18,20].

For health-related variables in this sample, lower depression scores were significantly associated with higher adherence for both adherence indicators. Other studies have similarly found that psychological health [11,20] is an important facilitator of adherence. Patients who had a CD4 count greater than 200 cells/uL, higher environment domain scores and better general health perception and overall quality of life scores reported higher adherence at their 6 month follow up. In a recent South African study, Wouters, Van Dammeb and Van Loon [42] found that baseline health (CD4 count) significantly influenced treatment outcomes during the first 6 months of ART. Patients with higher CD4 counts and better perceptions of their health are likely to have witnessed greater improvements in their health as a result of commencing ART. As Mills and colleagues meta-analysis indicates, this is likely to facilitate adherence.

Whilst the use of prayer predicted higher adherence in a Zambian study [20], the use of prayer was not associated with levels of adherence in the present study and was therefore excluded from analysis. The 'spirituality/religion/personal beliefs' domain contains items about whether the respondent considers their life to be meaningful, to what extent they are bothered about others blaming them for their illness, whether they fear for the future or worry about death and dying because of HIV. Higher scores (more positive attitudes about life and fewer worries about dying) in this domain were associated with lower adherence. These patients may have a lower perceived need for ART than other patients.

Behavioural variables associated with greater adherence include, high scores for IMB adherence information and behavioural skills and not using herbal medicines. Having greater knowledge about HIV and ARVs and greater HIV treatment behavioural skills and not using herbal medicines are known facilitators of adherence and confirm the IMB mdel [17-20,40]. Equally, higher social support scores and experiencing less discrimination were predictors of higher adherence in this study. Further research is needed to identify risk factors and to improve retention thorugh the use of social networks or emerging technologies for patients at risk for poor adherence [43].

## Limitations

This study also has limitations. For more than 95% of the patients studied viral loads were not available from medical records; they had not been done. So an important outcome of ART and ART adherence viral suppression could not be assessed. The patients who died or were lost to follow-up in the first 6 months were not included in the present study (selection bias). Some factors such as food insecurity, transportation barriers, and structural barriers of ARV adherence were not assessed [40]. Further, the assessment of ART adherence and other measures relied on self-report. However, there is increasing evidence indicating that adherence is reliably reported [41,44]. Caution is also urged in generalizing findings to other districts and provinces in the country. Investigation of factors related with long-term adherence would require longer follow-up than the current study.

## Conclusions

The adherence rate found in this study seems to be good. The use of two different adherence indicators was important for reducing bias through self-reporting and therefore enabling a greater potential range of determinants to be identified. Given the sample size and the large number of potential determinants of adherence in this study, variables were analysed in parsimonious subsets rather than one model. For the patients in this study, particularly those not living in urban areas, additional support may be needed to ensure patients are able to attend appointments or obtain their medications more easily. Adherence information and behavioural skills as part of the IMB model should be strengthened to improve adherence. Further psychological support is also required and patients' perceived need for ARTs should be routinely assessed. Although caution is urged in generalizing findings to other districts and provinces in the country, the results generally support the findings from other adherence studies in southern Africa.

## Competing interests

The authors declare that they have no competing interests.

## Authors' contributions

KP and NFDP conceptualized and designed the study, analysed and interpreted the data, drafted and revised the manuscript. SR participated in data collection, analysis and drafting of manuscript. JA participated in the design of the study and data analysis. All authors read and approved the final draft of the manuscript.

## Pre-publication history

The pre-publication history for this paper can be accessed here:

http://www.biomedcentral.com/1471-2458/10/111/prepub
